# Comparative effectiveness of home dialysis therapies: a matched cohort study

**DOI:** 10.1186/s40697-016-0105-x

**Published:** 2016-03-20

**Authors:** Gihad E. Nesrallah, Lihua Li, Rita S. Suri

**Affiliations:** The Li Ka Shing Knowledge Institute, Keenan Research Center, St. Michael’s Hospital, 30 Bond Street, Toronto, Ontario M5B 1W8 Canada; Nephrology Program, Humber River Regional Hospital, Toronto, Ontario Canada; Division of Nephrology, Western University, London, Ontario Canada; Centre de Recherche, Centre Hospitalier de l’Université de Montréal, Montréal, Québec Canada

## Abstract

**Background:**

Home dialysis is being increasingly promoted among patients with end-stage renal disease, but the comparative effectiveness of home hemodialysis and peritoneal dialysis is unknown.

**Objective:**

To determine whether patients receiving home daily hemodialysis have reduced mortality risk compared with matched patients receiving home peritoneal dialysis.

**Design:**

This study is an observational, propensity-matched, new-user cohort study.

**Setting:**

Linked electronic data were from the United States Renal Data System (USRDS) and a large dialysis provider’s database.

**Patients:**

The patients were adults receiving in-center hemodialysis in the USA between 2004 and 2011 and registered in the USRDS.

**Measurements:**

Baseline comorbidities, demographics, and outcomes for both groups were ascertained from the United States Renal Data System.

**Methods:**

We identified 3142 consecutive adult patients initiating home daily hemodialysis (≥5 days/week for ≥1.5 h/day) and matched 2688 of them by propensity score to 2688 contemporaneous US patients initiating home peritoneal dialysis. We used Cox regression to compare all-cause mortality between groups.

**Results:**

After matching, the two groups were well balanced on all baseline characteristics. Mean age was 51 years, 66 % were male, 72 % were white, and 29 % had diabetes. During 10,221 patient-years of follow-up, 1493/5336 patients died. There were significantly fewer deaths among patients receiving home daily hemodialysis than those receiving peritoneal dialysis (12.7 vs 16.7 deaths per 100 patient-years, respectively; hazard ratio (HR) 0.75; 95 % CI 0.68–0.82; *p* < 0.001). Similar results were noted with several different analytic methods and for all pre-specified subgroups.

**Limitations:**

We cannot exclude residual confounding in this observational study.

**Conclusions:**

Home daily hemodialysis was associated with lower mortality risk than home peritoneal dialysis.

**Electronic supplementary material:**

The online version of this article (doi:10.1186/s40697-016-0105-x) contains supplementary material, which is available to authorized users.

## ABRÉGÉ

**Mise en contexte:**

Les patients atteints d’insuffisance rénale terminale sont de plus en plus encouragés à pratiquer l’hémodialyse à domicile. Toutefois, l’efficacité de ce traitement par rapport à l’hémodialyse péritonéale est inconnue.

**Objectifs de l’étude:**

Cette étude visait à déterminer si le risque de mortalité des patients pratiquant l’hémodialyse quotidienne à domicile était inférieur à celui de patients ayant un profil similaire et recevant des traitements de dialyse péritonéale.

**Type d’étude:**

Il s’agit d’une étude observationnelle menée sur une cohorte de nouveaux utilisateurs, appariés par score de propension.

**Recrutement des participants:**

Les participants à l’étude, des adultes ayant reçu des traitements de dialyse en centre, aux États-Unis entre 2004 et 2011 et qui étaient inscrits dans les bases de données électroniques du *United States Renal Data System* (USRDS). Les données recueillies dans le USRDS ont été couplées aux bases de données d’un important fournisseur de produits de dialyse.

**Mesures:**

Le profil démographique des patients des deux groupes, ainsi que les renseignements cliniques au sujet des affections concomitantes et du pronostic inscrits au dossier au moment du recrutement, ont été établis à partir des données du USRDS.

**Méthodologie:**

Nous avons répertorié un total de 3142 patients qui amorçaient un programme de dialyse quotidienne à domicile, à raison d’un minimum de 1,5 heure par jour, au moins cinq jours par semaine. De ce nombre, 2688 ont été sélectionnés et appariés par score de propension à 2688 patients américains qui commençaient un programme de dialyse péritonéale à domicile au même moment. Le modèle de régression de Cox a été utilisé pour comparer les taux de mortalité toutes causes confondues dans les deux groupes.

**Résultats:**

Les patients des deux groupes étaient bien équilibrés après l’appariement quant à leurs caractéristiques initiales : ils étaient en majorité des hommes (66 %), blancs (72 %) dont l’âge moyen se situait à 51 ans. De plus, 29 % d’entre eux souffraient également de diabète. Au cours des 10 221 années-patients de suivi de l’étude, 1493 des 5336 participants sont décédés. On a noté un taux de mortalité significativement plus faible dans le groupe pratiquant la dialyse quotidienne à domicile par rapport au groupe recevant le traitement par dialyse péritonéale (12,7 vs 16,7 décès par 100 années-patients ; RR 0,75 ; 95 % IC 0,68 – 0,82 ; p < 0,001). Des résultats similaires ont été observés à l’aide de diverses méthodes d’analyse pour tous les sous-groupes prédéterminés.

**Limites de l’étude:**

Nous ne pouvons exclure que cette étude observationnelle contienne des variables confusionnelles résiduelles.

**Conclusions:**

Cette étude démontre que le risque de mortalité chez les patients qui pratiquent l’hémodialyse quotidienne à domicile est moindre que pour les patients qui subissent leur traitement par dialyse péritonéale.

## What was known before

Promoting home dialysis therapies is a major priority in high-income countries, yet studies directly comparing the effectiveness of home modalities are lacking. In a previous analysis, we found that home daily hemodialysis with low dialysate flow rates was associated with fewer hospitalizations and lower technique failure compared to peritoneal dialysis in the USA.

## What this adds

In this study, we found that home daily hemodialysis is associated with better survival than peritoneal dialysis. Patients considering home therapies, who are eligible for both modalities, should be informed of the possibility that outcomes with home daily hemodialysis are better than those with peritoneal dialysis.

## Background

The burden of end-stage renal disease (ESRD) in the USA is enormous. In 2011, ESRD patients comprised <1.1 % of Medicare beneficiaries, yet expended 6.2 % of the Medicare budget with costs of US$34.3 billion; an additional US$14.9 billion was spent on non-Medicare patients with ESRD [1, 2]. Although the majority of patients with ESRD receive in-center hemodialysis (HD), home dialysis modalities are being increasingly recommended as first-line renal replacement therapies [3–8]. Compared with in-center HD, home dialysis offers patients greater autonomy and improved quality of life at lower overall cost [9–11].

Peritoneal dialysis (PD) is the most widely utilized home dialysis therapy with prevalence rates of approximately 15 % in high-income countries, whereas home HD rates have typically lagged far behind at <2 % [12, 13]. However, with better predialysis education, increasing provider expertise, and dedicated home hemodialysis funding models, the last decade has seen rapid expansion of home HD, particularly in the form of daily treatments [14, 15]. New daily home HD devices feature prefilled dialysis solution bags and greater automation, thus bringing their technical complexity, accessibility, waste removal, and cost in line with that of PD.

In the absence of evidence comparing these two therapies, the choice between home HD and PD is predominantly determined currently by patient preference and predicated on an assumption of comparable outcomes with the various available therapies. However, recent funding reforms in the USA have increased the profitability of PD over home HD, creating a potential incentive for providers to promote this therapy over home HD [16–18]. This is evidenced by a recent surge in PD prescription in the USA [16]. With the increasing prevalence of home HD and availability of high-quality data, rigorous observational studies directly comparing home HD and PD have recently become feasible. Given these considerations, we conducted a matched cohort study to compare survival between home daily HD and PD.

## Methods

### Study design and setting

We conducted a retrospective cohort study comparing survival among patients receiving daily home HD and home PD in the USA. The Western University (London, Canada) Research Ethics Board approved the study.

### Data sources

The United States Renal Data System (USRDS) is a well-validated national database that includes data on demographics, diagnoses, biochemistry, dialysis claims, treatment history, hospitalizations, vital status, and causes of death (by *International Classification of Diseases, Ninth and Tenth Revisions* [ICD-9/10] codes) for all patients treated for ESRD in the USA since 1995 [19]. Although the USRDS can easily identify which patients are receiving PD, data on who is receiving home HD is not available. Thus, we identified USRDS patients who initiated home daily HD from January 2004 to October 2011 through a large US dialysis provider who maintains a comprehensive clinical database tracking detailed dialysis treatment data for all patients in its affiliated home dialysis units. Detailed dialysis prescription and treatment information for home daily HD patients was obtained from the provider’s database. To avoid information bias, we obtained all other data for both study cohorts (including demographics, comorbidity diagnoses, laboratory values, modality switches, and deaths) from the USRDS. Causes of death were grouped according to the 2012 USRDS Researcher’s Guide definitions [20]. The provider supplied data and allowed linkage to USRDS for a fee; neither the company nor the USRDS had any input into the study design, methods, analysis, or manuscript. All analyses adhered to a detailed, predefined protocol, and we prepared this manuscript according to Strengthening the Reporting of Observational studies in Epidemiology (STROBE) guidelines (Additional file 1) (30).

### Study sample

We included all consecutive adult patients (≥18 years) who initiated daily home HD through the large dialysis provider’s home dialysis facilities between January 2004 and October 2011. Home daily HD was defined as HD delivered in the patient’s home between 5 and 7 days per week, for 1.5 to 3.0 h per treatment. All home daily HD patients used a single dialysis device; >90 % received daily HD using low dialysate flows (<300 mL/min). We selected the comparator group of adults aged 18 years and older initiating PD for the first time and registered in the USRDS during the same time period. PD was defined as either *continuous cycler* (automated) or *continuous ambulatory* (manual) daily home PD, provided 7 days per week, using standard equipment. In order to restrict our cohorts to patients performing self- or partially assisted dialysis, we excluded residents of long-term care facilities and those with impaired mobility. Other exclusions are shown in Table 1.

**Table 1:** Cohort creation and distribution of follow-up time

Criterion or variable	Daily home HD	Home PD
Patients age ≥18 years, *n*	4509	208,086
Exclusions, *n* (%)		
Not in the time window^a^	1067 (23.7)	119,974 (57.7)
Nonindependent living	166 (3.7)	4355 (2.1)
Missing race	0 (0)	47 (0)
Missing comorbidity	0 (0)	8 (0)
Missing weight	55 (1.2)	6910 (3.3)
Albumin <1.0 or Hgb <5	25 (0.6)	754 (0.4)
Prior transplants >2	3 (0.1)	49 (0)
Follow-up <30 days	51 (1.1)	1067 (0.5)
Total excluded	1367 (30.3)	133,164 (64.0)
Eligible, *n* (%)	3142 (69.7)	74,922 (36.0)
Matched,^b^ *n* (%)	2668 (80.0)	2668 (3.6)
Follow-up time (years)		
Mean (SD)	1.9 (1.3)	2.0 (1.4)
Median (IQR)	1.7 (0.8–2.8)	1.7 (0.9–2.9)
Range	0.1–6.5	0.1–7.2

^a^The time window was defined as having started renal replacement therapy after 1995 and having started PD or daily HD between 2004 and 2011. Patients missing one or more of the following comorbidities were excluded: cancer, hypertension, congestive heart failure, cerebrovascular disease, peripheral vascular disease, chronic obstructive pulmonary disease, and diabetes

^b^The percentage matched was calculated as the proportion of eligible patients that were matched

### Primary outcome

The primary outcome was all-cause mortality, which is well validated against re-abstracted data in the USRDS (95 % agreement) [21]. The index date for both groups was defined as the first date of home HD or PD. To avoid survivor bias, patients were matched on the duration of survival with ESRD (eight categories) before the date of study enrollment.

### Statistical analyses

#### Derivation of propensity scores

We used logistic regression to calculate the probability of all included patients receiving daily home HD, conditional on variables that are known to be associated with either dialysis modality choice or survival on dialysis, or both [22]. Because daily HD and PD patients with similar propensity scores will have similar distributions of observed baseline variables, matching on the propensity score reduces the impact of selection bias [23].

#### Matching procedures

We used a “greedy” matching algorithm to match daily HD and PD patients by propensity score in a 1:1 ratio [24]. Because of their prognostic importance, we also matched on the duration of ESRD before the index date, year of initiation of renal replacement therapy, and age. We then iteratively tested various propensity score caliper widths and additional hard-matching variables until we achieved between-group standardized differences of <10 % for each variable, while maximizing the number of matched pairs [25]. Variables included in the final propensity score model are listed in Table 2.

**Table 2:** Baseline characteristics

Variable	Before matching (overall sample)			After matching		
	Home HD	PD	Standardized difference (%)	Home HD	PD	Standardized difference (%)
	*n* = 3142	*n* = 74,922		*n* = 2668	*n* = 2668	
Age (years), mean (SD)	50.0 (14.6)	56.3 (15.7)	41.6	51.3 (14.3)	51.4 (14.1)	0.3
18–29 years, %	9.4	6.2	12.1	7.5	7.5	0
30–39 years, %	17.2	10.2	20.6	15.7	15.4	0.9
40–49 years, %	20.2	15.8	11.5	19.8	19.9	0.1
50–59 years, %	24.8	22.8	4.7	25.7	26.7	2.2
60–69 years, %	19.2	23.1	9.5	21.1	20.5	1.5
70–79 years, %	7.8	16.3	26.4	8.7	8.9	0.9
>80 years, %	1.5	5.8	23.2	1.5	1.2	2.9
Sex (male), %	65.5	55.1	21.5	65.6	65.6	0
Race, %						
White	68.2	69.8	3.4	71.7	71.7	0
Black	28.1	23.6	10.2	25.9	25.9	0
Other	3.7	6.6	13.1	2.4	2.4	0
Smoking, %	6.9	6.7	0.7	7.0	7.5	1.9
Alcohol, %	0.5	0.7	1.9	0.6	0.8	3.1
Drugs, %	0.4	0.5	0.8	0.5	0.9	5.5
Private coverage, %	8.5	10.2	5.6	6.2	6.2	0
ESRD start date, %						
≤1976	0.1	0.1	0	0.1	0	1.3
1977–1994	0.6	1.0	4.1	0.5	0.6	2.6
1995–1999	5.8	1.6	21.3	3.2	3.1	0.5
2000–2004	12.6	5.7	24.3	9.6	10.8	4.2
2005–2009	74.9	73.5	3.2	80.1	79.5	1.5
≥2010	6.1	18.2	37.8	6.6	5.9	3.1
Duration of ESRD, mean (SD)	2.9 (3.0)	1.0 (2.5)	70.2	2.4 (2.4)	2.4 (2.4)	1.6
0–3 months, %	1.3	66.4	189.5	1.5	1.5	0
3–6 months, %	11.0	6.4	16.4	12.3	12.3	0
6–12 months, %	17.5	6.0	36.5	19.0	19.0	0
12–24 months, %	22.4	6.4	46.7	24.5	24.5	0
24–48 months, %	23.8	8.8	41.5	26.3	26.3	0
48–72 months, %	11.3	2.3	36.6	9.3	9.3	0
72–96 months, %	5.3	1.2	23.0	3.0	3.0	0
>96 months, %	7.4	2.6	22.4	4.0	4.0	0
Weight (kg), mean (SD)	90.5 (25.8)	82.3 (21.4)	34.4	88.5 (23.1)	88.4 (23.0)	0.1
Prior transplant, %						
0	92.9	97.0	18.9	94.7	94.8	0.1
1	6.4	2.7	17.9	4.8	4.9	0.1
2	0.7	0.3	5.7	0.5	0.4	1.3
Comorbidities, %						
Cancer	5.7	5.0	3.0	6.0	4.3	7.8
Hypertension	83.9	86.4	7.2	84.0	85.6	4.4
Congestive heart failure	17.4	19.9	6.5	16.2	16.2	0
Cerebrovascular disease	3.8	5.9	9.7	4.0	4.7	3.5
Peripheral vascular disease	7.0	9.8	10.1	7.2	9.1	7.1
Chronic obstructive pulmonary disease	4.3	4.9	3.0	4.3	4.8	2.4
Diabetes	27.3	31.8	9.7	28.8	28.8	0
Laboratory values, mean (SD)						
Albumin (g/dL)	3.3 (0.7)	3.4 (0.7)	15.6	3.3 (0.7)	3.2 (0.7)	13.9
Hemoglobin (g/dL)	10.1 (1.7)	10.4 (1.7)	18.2	10.1 (1.7)	10.0 (1.7)	9.8

Note: Except for age and duration of ESRD which are provided as of the index date (start of daily home HD or PD), all baseline variables are given as of the date of first renal replacement therapy (ESRD start date). Patients were matched by age (±5 years), gender, race, weight (±5 kg), duration of ESRD category, propensity score (0.02), ESRD start date (±5 years), congestive heart failure, diabetes, and medical coverage. The propensity score model included sex, race, weight, diabetes, hypertension, congestive heart failure, cerebrovascular disease, peripheral vascular disease, chronic obstructive pulmonary disease, cancer, number of prior transplants, medical coverage, history of smoking, and illicit drug use

#### Primary analysis

We followed patients until death, receipt of a kidney transplant, a maximum of 5 years after cohort entry, or to December 31, 2012 (last date of available records). In the primary analysis, we identified when patients changed their dialysis modality in follow-up, but did not censor the observation time for such events. This approach evaluated the potential long-term effects of initiating home daily HD versus PD. We used Cox regression stratified on matched sets to calculate hazard ratios (HR) with 95 % confidence intervals.

#### Sensitivity analyses

We repeated the primary analysis with censoring at the time of a modality switch (“as-treated” approach). Because a dialysis modality switch might herald failing health and an adverse event, we followed patients for an additional 90 days after switching, attributing deaths to the baseline exposure. We also repeated the main analysis using the entire sample, with adjustment for propensity score and with inverse probability of treatment weighting using the propensity score [26]. Finally, because comorbid conditions are known to be underreported in the USRDS at the initiation of chronic dialysis [27], we used hospitalization claims data for Medicare beneficiaries to identify comorbid conditions that accumulated between the first ESRD service date and the date of initiation of home daily HD or PD.

#### Subgroup analyses

We repeated the primary analysis for the following predefined subgroups using the median value in the daily HD group as the cut-point for continuous variables: age, weight, duration of ESRD prior to home dialysis initiation, vascular access type, diabetes, and congestive heart failure. We also created subgroups according to propensity score quintiles (defined by the daily HD group) and tested for an interaction between propensity score quintile and exposure. This allowed us to assess the effect of daily HD on survival, even among patients least likely to receive home daily HD.

#### Assessing the potential impact of unmeasured confounding

We adapted the method of Lin et al. [28] for use with matched cohorts to evaluate the potential impact of an unmeasured confounder (“*U*”) on the primary treatment effect estimate. Briefly, we iteratively examined a range of scenarios in which the prevalent rate of *U* ranged between 1 % and 100 % in the PD cohort and calculated the hazard ratio associated with *U* that would result in the 95 % confidence interval of the association between dialysis modality and mortality to include unity (1.0). We generated a family of curves for prevalence rates of *U* in the daily HD cohort of 5, 10, 20, and 40 %. We used Cox regression with a robust covariance estimator to derive the hazard ratio associated with the confounder.

All tests of statistical significance were two-tailed. We interpreted a *P* ≤ 0.05 as being significant, although *P* ≤ 0.10 was interpreted as potentially significant for interaction tests. All analyses were performed in SAS version 9.3 (SAS Institute, Cary, NC).

## Results

### Study sample, baseline characteristics, and dialysis prescriptions

Of 3142 eligible home daily HD patients, 2668 (85 %) were matched to a patient receiving PD (Table 1). After matching, baseline variables were balanced between groups, with all standardized differences <10 % (Table 2). In all, 94 % were Medicare beneficiaries. Access types in home daily HD patients were catheters (50 %), fistulae (18 %), grafts (2 %), and unknown (30 %). At baseline, home HD patients received a mean 2.7 ± 0.6 h per treatment session and treatment time remained constant throughout study follow-up (Additional file 1: Figure S1).

### Mortality

**Fig. 1:**
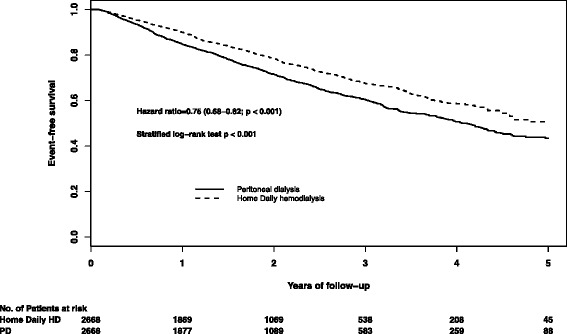
Kaplan-Meier survival curve of home daily hemodialysis versus peritoneal dialysis

A total of 1493 of 5336 patients died over 10,221 person-years (mean follow-up 1.9 ± 1.4 years; median 1.7, range 0.2–7.2 years). The mortality rate was 16.7 deaths per 100 patient-years for patients receiving PD compared to 12.7 deaths per 100 patient-years for patients receiving home daily HD (HR 0.75; 95 % CI 0.68–0.82; *P* < 0.001) (Fig. 1, Table 3). Causes of death were similar between groups, with cardiovascular and infection-related causes accounting for >50 % of deaths (Table 4).

**Table 3:** Hazard ratios for all-cause mortality in primary and sensitivity analyses

Group	Number of patients	Number of events^a^	Deaths per 100 person-years	HR (95 % CI)^b^	*P* value
Main analysis					
Matched sample, intent-to-treat analysis					
PD patients (referent)	2668	868	16.71	1	<0.001
Daily HD patients	2668	625	12.56	0.75 (0.68–0.82)	
Additional analyses					
Unmatched sample, intent-to-treat analysis, inverse probability of treatment weighting					
PD patients (referent)	74,922	24,638	14.99	1	<0.001
Daily HD patients	3142	719	12.20	0.84 (0.82–0.86)	
Matched sample, as-treated analysis (90-day follow-up after modality switches)					
PD patients (referent)	2668	410	13.33	1	0.005
Daily HD patients	2668	496	11.52	0.83 (0.74–0.95)	
Unmatched sample, intent-to-treat analysis, adjusting for propensity score					
PD patients (referent)	74,922	24,638	14.99	1	0.002
Daily HD patients	3142	719	12.20	0.89 (0.82–0.96)	
Matched sample, intent-to-treat analysis, index date within 12 months of ESRD date					
PD patients (referent)	877	280	17.04	1	<0.001
Daily HD patients	877	174	11.03	0.65 (0.54–0.78)	

^a^Follow-up truncated at 5 years.

^b^Hazard ratio was derived from Cox proportional hazards model including robust variance estimator to account for within-pair correlation

**Table 4:** Causes of death

Cause of death	Home daily HD, *n* (%)	PD, *n* (%)
	*n* = 2668	*n* = 2668
Total	630 (23.6)	874 (32.8)
Cardiovascular	259 (41.1)	372 (42.6)
Infection	82 (13.0)	118 (13.5)
Withdrawal from dialysis	58 (9.2)	86 (9.8)
Bleeding	10 (1.6)	9 (1.0)
Other	89 (14.1)	119 (13.6)
Missing	132 (21.0)	170 (19.5)

### Modality switches

During follow-up, 2 % (39 of 2688) of daily patients switched to PD, whereas 20 % (531 of 2688) of PD patients switched to home daily HD; of the latter, 168 of 531 patients received home daily HD for ≤30 days. Further, 19 % (507 of 2688) daily HD patients and 39 % (1040 of 2688) PD patients discontinued home therapy, switching to in-center conventional HD (*P* = 0.002). When we repeated the primary analysis censoring for modality switches at 90 days, the HR of death with home daily HD compared to PD was 0.83 (95 % CI 0.74–0.95; *P* = 0.005).

### Additional analyses

**Fig. 2:**
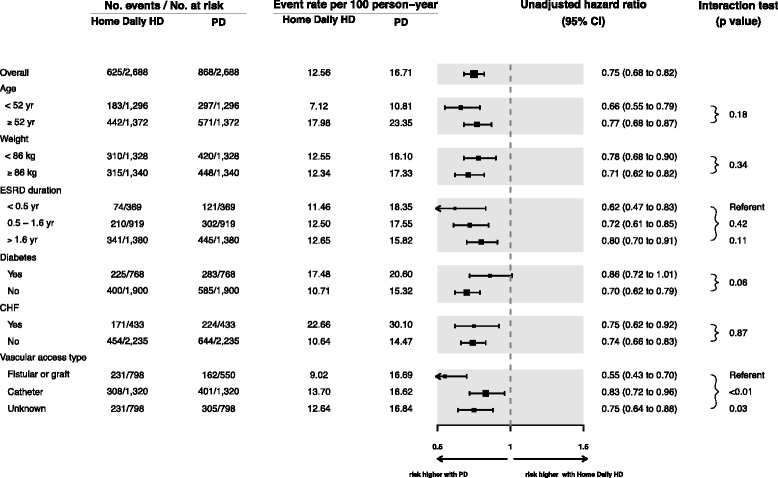
Subgroup analyses for matched cohorts. Cut-points for age and weight are based on median values in the home daily HD group. The cut-points for ESRD duration are based on a pragmatic definition of “early” initiation (6 months) and the median time with ESRD before study entry in the home daily HD group. *CHF* congestive heart failure, *DHD* daily home hemodialysis, *PD* peritoneal dialysis

When we analyzed the entire sample (both incident and prevalent patients) with adjustment for the propensity score and inverse probability of treatment weighting, we obtained similar results as for the primary analysis (Table 3). Daily HD was associated with better survival compared to PD for all prespecified subgroups up to 5 years (Fig. 2, and Additional file 1: Table S1). Interaction *P* values for diabetes and vascular access type were ≤0.10, suggesting greater benefit associated with daily HD among those with arteriovenous access (fistulae and grafts) and those without diabetes. Daily HD was associated with similar benefit across subgroups defined according to propensity score quintile, suggesting that survival was better with daily HD even among patients whose propensity scores were most consistent with selection of PD. When we supplemented baseline comorbidity data in the matched cohorts with diagnoses that accrued between the first ESRD service date and the initiation of home HD and PD (Additional file 1: Table S2), all standardized differences remained below 10 %, confirming adequate balance of baseline prognostic variables in the main analyses.

### Potential effects of residual confounding

**Fig. 3:**
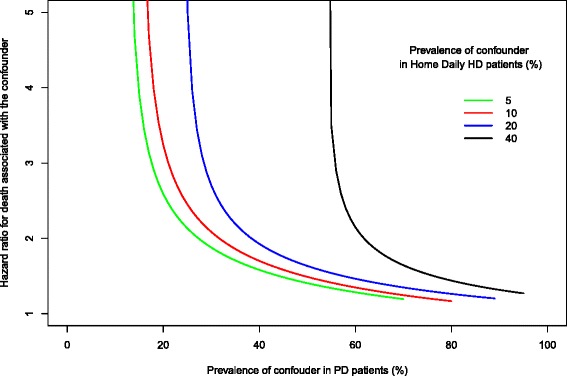
Effect of unmeasured confounding. This sensitivity analysis illustrates how powerful a single unmeasured confounder (e.g., “frailty”) would have to be to account for the survival advantage of daily home HD over PD. The *y*-axis denotes the hazard ratio that would have to be associated with the unmeasured confounder to raise the upper limit of the 95 % CI of the primary analysis effect estimate to include unity (1.0, no association). For example, if the prevalence of the unmeasured confounder was 5 % in the daily HD group and 20 % in the PD group, a hazard ratio of 2.63 associated with the confounder would be required to account for the observed advantage of daily HD over PD in the main analysis (see text)

We considered a range of scenarios in which we varied the prevalence of a hypothetical unmeasured confounder (*U*; e.g., “frailty”)[29] from 0 to 100 % in the PD cohort. In Fig. 3, the prevalence of *U* in the PD cohort is plotted on the *x*-axis, the hazard ratio for death associated with *U* is plotted on the *y*-axis, and the prevalence of *U* in the daily HD group is represented by a family of curves. From Fig. 3, it can be seen that if the prevalence of the missed confounder *U* was 5 % in the daily HD group (green curve), the prevalence of *U* in the PD group would have to be at least 20 % in order for the upper limit of the 95 % confidence interval observed in our primary analysis to be raised from 0.82 to 1.0 (i.e., indicating no significant association between daily HD and mortality) assuming that the hazard ratio of mortality associated with *U* was 2.6. If the hazard ratio of mortality associated with *U* was only 1.9, the prevalence of *U* in the PD group would have to be at least 30 %. Alternatively, if the prevalence of *U* in the PD group was low (<10 %), the hazard ratio of death associated with *U* would have to approach infinity. The other curves show that as the assumed prevalence of *U* in the daily group rises, the prevalence of *U* in the PD group would have to be even higher to account for the survival difference between daily HD and PD observed in our analysis.

## Discussion

In this matched, observational comparative effectiveness study, we found that among prevalent adult patients with ESRD receiving dialysis at home, home daily HD was associated with a moderate survival benefit compared with PD. The association persisted across multiple analyses, prespecified subgroups, and for all causes of death including infection and cardiovascular disease. In our subgroup analysis, the survival advantage associated with daily home HD improved in a graded manner with earlier conversion to home dialysis.

In most high-income countries, dialysis programs are encouraged to promote a “home-first” approach to modality selection [3, 5, 15, 30, 31]. This is justified by the observation that patients receiving home dialysis in the form of either PD or HD have better survival than those who undergo treatment in-center [32–34]. However, it is unclear whether the better outcomes observed with home therapies are due to the home modalities themselves or to the selection of healthier patients for home dialysis. There has been minimal data actually comparing the different home dialysis therapies with one another. Recently, Nadeau-Fredette et al. [35] found a strong survival advantage associated with home HD compared with PD among patients initiating home dialysis in Australia and New Zealand. This study included only incident dialysis-naïve patients, thus potentially informing the question of initial dialysis modality choice. However, the generalizability of these findings outside of Australia and New Zealand may be limited because many patients received long nocturnal HD and all used standard HD equipment. In the USA, the most prevalent form of home HD is with devices that use low dialysate flow rates as evaluated in our study. Moreover, it is unclear if the Nadeau-Fredette et al. findings are the result of better outcomes with home HD or worse outcomes with PD. In North America, PD has been consistently associated with similar or better survival than in-center conventional HD [36, 37], whereas comparative survival studies from Australia and New Zealand have shown the opposite [34]. To our knowledge, ours is the first study to directly address the comparative effectiveness of the two most prevalent forms of home dialysis in the USA and informs decision-making for the growing number of patients who initiate in-center HD and then switch to a home modality.

There are several biologically plausible reasons by which home daily HD may confer a survival advantage over PD. We found that more than 40 % of deaths were cardiovascular disease-related. In the recent Frequent Hemodialysis Network Daily Trial, in-center daily HD resulted in greater small solute clearance, improved phosphate control, reduced extracellular fluid volume, and regression of left ventricular hypertrophy compared to conventional HD [38]. We would expect similar physiological benefits with home daily HD. The second most common cause of death was infection. Although 100 % of PD patients have an intraperitoneal catheter, almost one-fifth of home HD patients in our cohort had native vessel fistulae. Because arteriovenous fistulae have substantially lower infection rates than PD catheters, this may partially explain the fewer infection-related deaths with daily HD. In addition, given that uremia has been linked to impaired immunity [39, 40], it is conceivable that better clearance of uremic wastes afforded by daily HD contribute to fewer infection-related deaths. Finally, almost 10 % of deaths were related to dialysis withdrawal. We found that patients receiving PD were more likely to discontinue home therapy and return to in-center HD than patients receiving home HD. To what extent loss of autonomy and having to stop home therapy may have contributed to more deaths from dialysis withdrawal with PD is unclear.

Drawing on the same data sources and similar methods as used in this study, we recently also compared hospitalization events among patients undergoing home daily HD and PD [41]. Compared with PD, daily home HD was associated with a lower rate of hospitalizations (HR 0.73; 95%CI 0.67–0.79; *P* < 0.001) and fewer hospital days (5.2 versus 9.2 days/patient-year; *P* < 0.001). In that study, we also found the rate of permanent failure of daily home HD to be lower than that of PD with 15 % versus 63 % of patients switching to in-center HD during the first 2 years of follow-up. Concordant results across these related outcomes increase confidence in our observed effect estimate for survival.

Our study has several strengths. Our comparison of two home-treated populations reduces the risk of confounding due to factors often associated with self-care ability and the home treatment setting. In contrast, most prior work has focused on comparisons between home and in-center therapies [33, 36, 42–44]. Home and in-center dialysis recipients likely differ systematically across a range of unmeasured variables that are, in turn, associated with mortality, including health literacy [45], emotional well-being [46], income [47], and cognitive function [48]. Although not directly measured, these factors are more likely to be balanced between our two home dialysis cohorts than in studies comparing home with in-center dialysis patients. We analyzed data from a complete dataset of consecutive patients receiving daily home HD from a single provider, eliminating bias that arises from inclusion of only prevalent long-term survivors. We obtained baseline variables and outcomes data from a single well-validated data source to avoid information bias. We used rigorous methods to match patients on all known baseline characteristics. We “hard matched” more than eight different categories of the duration of ESRD—an extremely important prognostic variable that is often not addressed adequately in other matched observational studies—and we matched on the year of ESRD onset to address era effects.

Despite propensity score matching, residual confounding remains a potential explanation for our findings. To address the potential impact of this, we performed a bias analysis. We found that in order for (an) unmeasured confounding factor(s) to completely explain the observed association between home daily HD and survival, either the hazard of death due to the confounding factor would have to be extremely high or the prevalence of the confounding factor would have to be implausibly high (Fig. 3). It is rare in the literature that even the most important risk factors are associated with hazard ratios >2 (e.g., smoking) [49]. It is also unlikely with the matching procedures we used that the prevalence of any residual confounding factor would be as high as 20 % in the PD group and only 5 % in the home daily HD group. Nevertheless, we cannot exclude the combined effects of multiple unmeasured confounders and the inclusion of two home-treated populations in this study may not have mitigated all bias due to factors such as socioeconomic status, presence of a caregiver, cognitive ability, or self-efficacy.

We recognize other limitations of our study. First, we do not know reasons for modality switches nor what impact these may have had on the observed mortality rates; historically, this has been a major limitation in other studies comparing PD with in-center HD [33, 44, 50–52]. Prospective data characterizing reasons for modality switches and initial modality choices would provide useful prognostic information for inclusion in future comparative studies and should be collected by renal registries and dialysis provider databases. It is reassuring that the hazard ratios did not change appreciably when we censored for modality switches. Second, our matched cohort consisted largely of prevalent patients (i.e., >3 months with ESRD before starting home dialysis). Although our subgroup analyses examining patients who started PD or home HD within 3 months of ESRD showed better survival with daily HD, this subgroup represented <2 % of the total cohort; thus, our results may not be applicable to patients initiating dialysis for the first time. Third, our comorbidity information was ascertained at the initiation of ESRD rather than at the initiation of PD or home HD. However, it should be noted that when we supplemented baseline covariate data with hospitalization diagnoses, the prevalence rates of these conditions did not change significantly and the groups remained well balanced. This strongly suggests that the accumulation of comorbidities over time was not a major source of confounding. Fourth, we had no data describing residual renal function, which is strongly associated with improved survival. However, this very plausible potential source of bias further increases our confidence in our effect estimates because residual function is usually better preserved with PD than with HD. Finally, our findings may not be generalizable outside of the USA or to other large dialysis organizations operating in the USA. These considerations notwithstanding, we recognize that the possibility of residual confounding can never be completely eliminated from observational studies. Given recent challenges in recruiting patients in clinical trials comparing dialysis therapies [53], such studies may not be available in the foreseeable future. Therefore, our study provides the best possible estimate of effect with available data and methodological approaches.

## Conclusions

Our findings may be of interest to several decision makers. In the USA, the recent adoption of the prospective (bundled) payment system has made PD more profitable [18] and this has been accompanied by unprecedented growth in PD prescription in for-profit facilities [16]. A European expert panel recently suggested a “modality neutral” approach to educating patients on dialysis options; in this paradigm, providers would suggest that home HD and PD provide similar survival outcomes and that patients’ choices should be based on other values and preferences [8]. Our findings challenge the appropriateness of current approaches to modality selection, particularly because many patients place a high value on survival when choosing dialysis modalities [54]. Nevertheless, although our study addresses mortality in one cohort, it is by no means definitive. Additional well-conducted studies evaluating patient-important outcomes, including quality of life, technique sustainability, hospitalizations, and mortality in other cohorts of home HD and PD patients, are needed before making widespread recommendations regarding the “optimal first choice” therapy. Full disclosure of the potential benefits and harms of all available dialysis modalities should be provided to all patients before they select a therapy. While awaiting more definitive comparative effectiveness studies, patients eligible for home HD and PD should be made aware of the potentially greater survival associated with home HD suggested in this observational study.

## Additional file

Additional file 1: Supplementary materials. (DOCX 52 kb)

## Abbreviations

ESRD: end-stage renal disease
HD: hemodialysis
HR: hazard ratio
PD: peritoneal dialysis
USRDS: United States Renal Data System
