# Supplementary material for: Comparative effectiveness of home dialysis therapies: a matched cohort study
Source: Can J Kidney Health Dis. 2016 Mar 20;3:19. doi: 10.1186/s40697-016-0105-x (PMC4802626; doi:10.1186/s40697-016-0105-x)
Supplement: Supplementary file 1 — Supplementary materials. (DOCX 52 kb) [file 40697_2016_105_MOESM1_ESM.docx]

**SUPPLEMENTARY MATERIALS**

**Figure S1: Treatment Time Received by Home Daily Hemodialysis Patients Over Time**

**Table S1: Details of Subgroup Analyses for Matched Cohorts**

| **Subgroup** | **No. of Patients** | | **No. of Events** | | **Event rate (per 100 person-yrs)** | | **Hazard Ratio**  **(95% CI)** | **p-value for interaction** |
| --- | --- | --- | --- | --- | --- | --- | --- | --- |
|  | **DHD** | **PD** | **DHD** | **PD** | **DHD** | **PD** |  |  |
| Overall | 2688 | 2688 | 625 | 868 | 12.56 | 16.71 | 0.75(0.68-0.82) |  |
| Duration of end-stage renal disease duration before index date* |  |  |  |  |  |  |  |  |
| < 6 months | 369 | 369 | 74 | 121 | 11.46 | 18.35 | 0.62 (0.47-0.83) | - |
| 6 months - 1.6 yrs | 919 | 919 | 210 | 302 | 12.50 | 17.55 | 0.72(0.61-0.85) | 0.416 |
| > 1.6 yrs | 1380 | 1380 | 341 | 445 | 12.65 | 15.82 | 0.80 (0.70-0.91) | 0.112 |
| Age^*^ |  |  |  |  |  |  |  |  |
| <52 years | 1296 | 1296 | 183 | 297 | 7.12 | 10.81 | 0.66 (0.55-0.79) | - |
| ≥52 years | 1372 | 1372 | 442 | 571 | 17.98 | 23.35 | 0.77 (0.68-0.87) | 0.184 |
| Weight^*^ |  |  |  |  |  |  |  |  |
| < 86 kg | 1328 | 1328 | 310 | 420 | 12.55 | 16.10 | 0.78 (0.68-0.90) | - |
| ≥ 86 kg | 1340 | 1340 | 315 | 448 | 12.34 | 17.33 | 0.71(0.62-0.82) | 0.343 |
| Diabetes |  |  |  |  |  |  |  |  |
| No | 1900 | 1900 | 400 | 585 | 10.71 | 15.32 | 0.70 (0.62-0.79) | - |
| Yes | 768 | 768 | 225 | 283 | 17.48 | 20.60 | 0.86 (0.72-1.01) | 0.058 |
| Congestive heart failure |  |  |  |  |  |  |  |  |
| No | 2235 | 2235 | 454 | 644 | 10.64 | 14.47 | 0.74 (0.66-0.83) | - |
| Yes | 433 | 433 | 171 | 224 | 22.66 | 30.10 | 0.75 (0.62-0.92) | 0.865 |
| Vascular access type |  |  |  |  |  |  |  |  |
| Fistula or Graft | 550 | 550 | 86 | 162 | 9.02 | 16.69 | 0.55(0.43-0.70) | - |
| Catheter | 1320 | 1320 | 308 | 401 | 13.70 | 16.62 | 0.83(0.72-0.96) | 0.003 |
| Unknown | 798 | 798 | 231 | 305 | 12.64 | 16.84 | 0.75(0.64-0.88) | 0.028 |
| Propensity score |  |  |  |  |  |  |  |  |
| Quintile 1 | 534 | 534 | 142 | 186 | 14.17 | 18.35 | 0.77 (0.63-0.94) | - |
| Quintile 2 | 534 | 534 | 118 | 171 | 12.18 | 17.81 | 0.68 (0.55-0.85) | 0.410 |
| Quintile 3 | 533 | 533 | 122 | 191 | 12.63 | 18.31 | 0.69 (0.56-0.86) | 0.468 |
| Quintile 4 | 534 | 534 | 125 | 156 | 11.84 | 14.14 | 0.84 (0.67-1.05) | 0.590 |
| Quintile 5 | 533 | 533 | 118 | 164 | 11.40 | 15.28 | 0.75 (0.60-0.94) | 0.838 |

^*^Categories defined by median value in home daily HD group.

**Table S2: Comorbid Conditions Supplemented with Hospitalization Diagnostic Codes Reported between First ESRD Service Data and Index Date**

| Comorbid condition | After Matching | | |
| --- | --- | --- | --- |
|  | Home HD  N=2668 | PD  N=2668 | Standardized difference (%) |
| Cancer | 9.1 | 7.0 | 7.1 |
| Hypertension | 85.6 | 86.8 | 3.4 |
| Congestive heart failure | 29.7 | 30.7 | 2.1 |
| Cerebrovascular disease | 8.3 | 9.0 | 2.4 |
| Peripheral vascular disease | 12.4 | 14.8 | 7.0 |
| Chronic obstructive pulmonary disease | 13.8 | 14.3 | 1.5 |
| Diabetes | 34.1 | 38.4 | 9.1 |
| Ischemic heart disease | 17.4 | 19.8 | 6.2 |

**APPENDIX: STROBE CHECKLIST**

|  | | Item No | | Recommendation | Location in Report |
| --- | --- | --- | --- | --- | --- |
| Title and abstract | | 1 | | (a) Indicate the study’s design with a commonly used term in the title or the abstract | Title page |
|  |  |  |  | (b) Provide in the abstract an informative and balanced summary of what was done and what was found | Abstract |
| Introduction | | | | |  |
| Background/rationale | | 2 | | Explain the scientific background and rationale for the investigation being reported | Introduction |
| Objectives | | 3 | | State specific objectives, including any pre-specified hypotheses | Introduction |
| Methods | | | | |  |
| Study design | | 4 | | Present key elements of study design early in the paper | Study Design and Setting |
| Setting | | 5 | | Describe the setting, locations, and relevant dates, including periods of recruitment, exposure, follow-up, and data collection | Study Design and Setting |
| Participants | | 6 | | Give the eligibility criteria, and the sources and methods of selection of participants. Describe methods of follow-up | Study Populations and Home Dialysis Therapies |
| Variables | | 7 | | Clearly define all outcomes, exposures, predictors, potential confounders, and effect modifiers. Give diagnostic criteria, if applicable | Data Sources |
| Data sources/ measurement | | 8 | | For each variable of interest, give sources of data and details of methods of assessment (measurement). Describe comparability of assessment methods if there is more than one group | Data Sources |
| Bias | | 9 | | Describe any efforts to address potential sources of bias | Derivation of Propensity Scores  Assessing the Potential Impact of Unmeasured Confounding |
| Study size | | 10 | | Explain how the study size was arrived at | NA |
| Quantitative variables | | 11 | | Explain how quantitative variables were handled in the analyses. If applicable, describe which groupings were chosen and why | Data Sources |
| Statistical methods | | 12 | | (a) Describe all statistical methods, including those used to control for confounding | Statistical Analyses |
|  |  |  |  | (b) Describe any methods used to examine subgroups and interactions | Subgroup Analyses |
|  |  |  |  | (c) Explain how missing data were addressed | N/A |
|  |  |  |  | (d) If applicable, explain how loss to follow-up was addressed | Primary Analysis |
|  |  |  |  | (e) Describe any sensitivity analyses | Sensitivity Analyses |
| Results | | | | |  |
| Participants | 13 | | (a) Report numbers of individuals at each stage of study—eg numbers potentially eligible, examined for eligibility, confirmed eligible, included in the study, completing follow-up, and analyzed | | Study Sample, Baseline Characteristics and Dialysis Prescriptions |
|  |  |  | (b) Give reasons for non-participation at each stage | | N/A |
|  |  |  | (c) Consider use of a flow diagram | | Appendix |
| Descriptive data | 14 | | (a) Give characteristics of study participants (eg demographic, clinical, social) and information on exposures and potential confounders | | Table 1 |
|  |  |  | (b) Indicate number of participants with missing data for each variable of interest | | Table 1 |
|  |  |  | (c) Summarize follow-up time (eg, average and total amount) | | Competing Events; Mortality |
| Outcome data | 15 | | Report numbers of outcome events or summary measures over time | | Competing Events; Mortality |
| Main results | 16 | | (a) Give unadjusted estimates and, if applicable, confounder-adjusted estimates and their precision (eg, 95% confidence interval). Make clear which confounders were adjusted for and why they were included | | Mortality |
|  |  |  | (b) Report category boundaries when continuous variables were categorized | | Mortality |
| Other analyses | 17 | | Report other analyses done—eg analyses of subgroups and interactions, and sensitivity analyses | | Sensitivity Analyses; Subgroup Analyses |
| Discussion | | | | |  |
| Key results | 18 | | Summarize key results with reference to study objectives | | Discussion |
| Limitations | 19 | | Discuss limitations of the study, taking into account sources of potential bias or imprecision. Discuss both direction and magnitude of any potential bias | | Discussion |
| Interpretation | 20 | | Give a cautious overall interpretation of results considering objectives, limitations, multiplicity of analyses, results from similar studies, and other relevant evidence | | Discussion |
| Generalizability | 21 | | Discuss the generalizability (external validity) of the study results | | Discussion |
| Other information | | | | |  |
| Funding | 22 | | Give the source of funding and the role of the funders for the present study and, if applicable, for the original study on which the present article is based | | Funding |
